# The molecular architecture of a complex social behavior: gregarious song

**DOI:** 10.1186/s12868-023-00833-0

**Published:** 2023-11-02

**Authors:** Tyler J. Stevenson

**Affiliations:** School of Biodiversity, One Health and Veterinary Medicine, University of Glasgow, Glasgow, United Kingdom

**Keywords:** Season, Birdsong, GnRH, Testosterone, Illumina, Photoperiod, Social, GLIN1, DRD2

## Abstract

The medial preoptic area (mPOA) regulates the probability and intensity of singing behavior in birds. Polzin and colleagues examined the molecular changes in the mPOA that were associated with gregarious song in European starlings (*Sturnus vulgaris*). High-throughput transcriptome analyses identified glutamate and dopamine pathways were highly enriched with gregarious song.

## Main text

The neuroanatomical basis of social behavior is well described [1]. A series of discrete cortical, limbic, and hypothalamic nuclei function to facilitate, or inhibit a wide range of complex behaviors across vertebrates. Over the past two decades, significant advances into the cellular and molecular basis of social behavior have yielded substantial gains on the role of specific neuropeptides, such as vasopressin and oxytocin for the control of social behavior [2]. However, most molecular research has used a limited number of mammalian species due to the relative ease in which nucleic acid information could be derived from biomedical models, (i.e., mice). Recent advances in genome sequencing and large-scale collaborations such as the Avian Phylogenomics Project, have permitted the ability to develop well characterised genome assemblies and annotations [3]. These Projects are now facilitating the molecular dissection of complex social behaviors from non-traditional animal models.

**Fig. 1:**
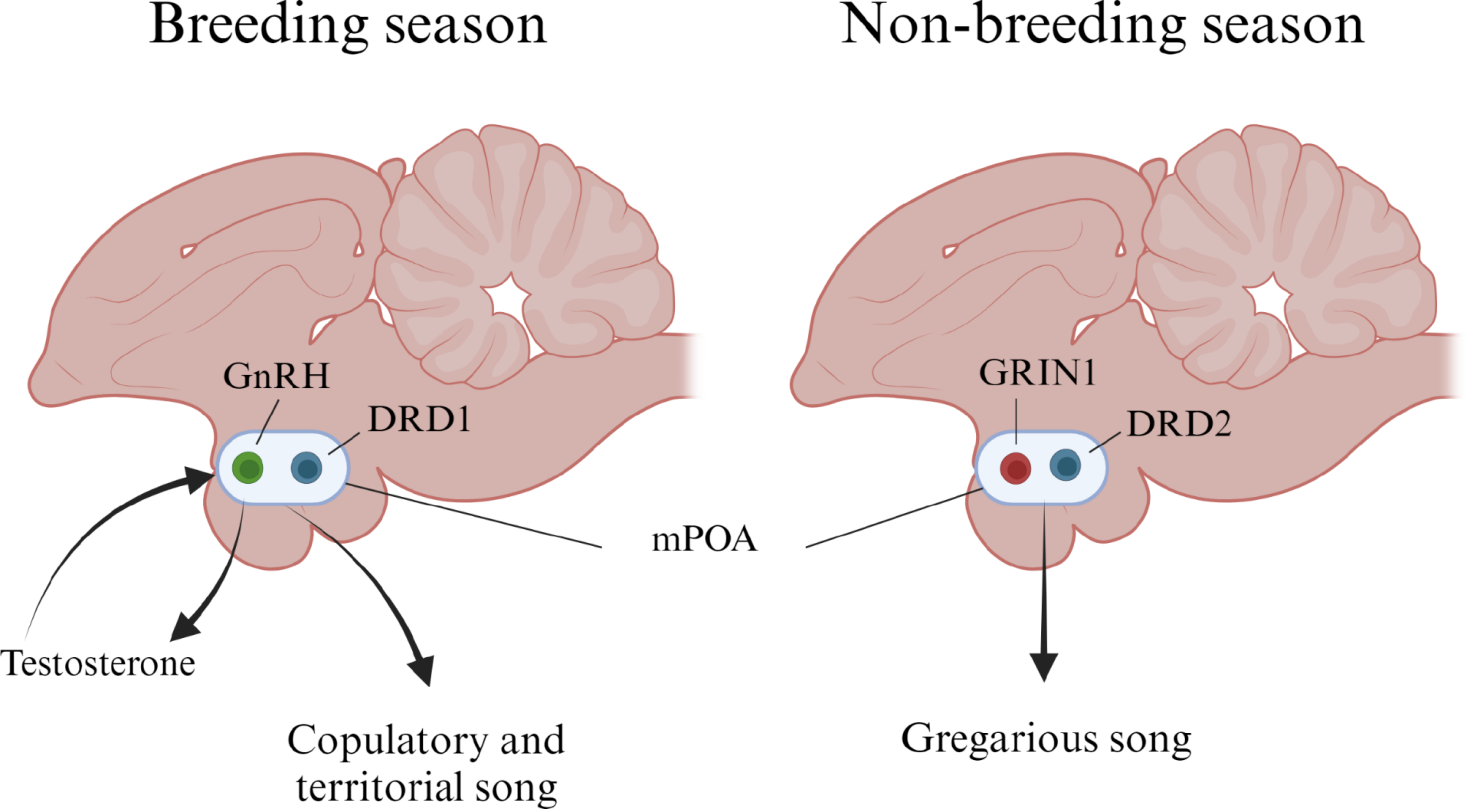
Medial preoptic area drives seasonally contextual singing behavior in birds. Gonadotropin-releasing hormone (GnRH) expression in the medial preoptic area (mPOA) is elevated in breeding birds. GnRH governs seasonal changes in testosterone production which facilitates sexual behavior, as well as territorial and copulatory singing behavior. Other neurochemical systems, such as dopamine, act to increase the motivation and reward properties associated with singing. Conversely, gregarious song production during the non-breeding season was found to be highly associated with dopamine pathways as well as glutamate signalling systems. Abbreviations: Dopamine receptor (DRD); Glutamate ionotropic receptor N-methyl-D-aspartate type subunit 1 (GRIN1)

Songbirds, in the order Passeriformes, produce complex, learned vocalization called song [4]. A well described neural circuit is known to be involved in song learning and song production. Yet, the motivation for the bird to sing is driven by the preoptic area of the hypothalamus, a critical node in the social behavior circuit [5]. In most songbird species, the male bird sings at high levels during the breeding season to defend territories and attract potential mates [6]. Previous work had established that lesions to the preoptic area eliminated singing behavior in male starlings (*Sturnus vulgaris*) [7]. Complementary DNA (cDNA) microarray analyses of the preoptic area in male starlings demonstrated that multiple molecular and cellular changes occur across the seasons [8] and that gonadotropin-releasing hormone (GnRH) neurons are one population that serves to control the timing of breeding and non-breeding states [9]. Yet in male canaries, the number of songs, duration of song and types of song syllables were negatively correlated with GnRH cells [10]. Higher GnRH in the breeding season stimulates the synthesis of testosterone production which provides feedback into the medial preoptic area (mPOA) to increase the motivation for singing [11]. Testosterone acts on other neurochemical substrates such as dopamine receptor expressing cells [12] to increase the motivation to sing copulatory and territorial song during the breeding season (Fig. 1). During the non-breeding season, both male and female birds sing and produce ‘gregarious’ song that functions to maintain social cohesion despite low levels of GnRH and testosterone. Our understanding of the molecular basis for the motivation of birds to engage in gregarious singing behavior is poorly understood.

The publication by Polzin and colleagues [13] has now characterised the molecular basis of the motivation for gregarious song. Using semi-natural conditions, the researchers monitored singing behavior from group housed male and female starlings over five days. The experimental design was sufficient to classify high- or low-gregarious song production in individual birds. Thirty minutes after individual singing bouts and the mPOA of the hypothalamus was dissected. The authors prepared RNA-sequencing libraries to assay genome-wide gene expression profiles. Many transcripts were observed to be differentially expressed between high and low gregarious song and several were associated with key neurotransmitter pathways. Specifically, birds singing high levels of gregarious song were found to exhibit greater expression levels of dopamine receptors (i.e., *DRD2, DRD5*), gamma-aminobutyric acid (GABA) receptor subunits (i.e., *GABRB3, GABRB5, GABRD*), glutamate metabotropic receptor 5 (*GRM5*), glutamate ionotropic receptor kainite type (*GRIK*) and alpha-amino-3-hydroxy-5-methyl-4-isoxazole propionate (AMPA) type (GRIA) subunits (i.e., *GRIK3*, *GRIA1*, *GRIA2*), muscarinic acetylcholine receptor 4 (*CHRM4*) and choline O-acetyltransferase (*CHAT*). The monoamine transporter (i.e., *SLC18A2*) was found to be expressed at higher levels in low singing birds. There were very similar expression profiles in high singing males and females suggesting a lack of sex-difference in the molecular basis for the motivation to produce gregarious song. Then, using a multiscale embedded gene co-expression network analysis (MEGENA) the authors identified that hundreds of nested modules and several were significantly correlated with singing condition. By focusing on a subset of modules, the authors again identified glutamate pathways were enriched including multiple glutamate receptor genes (*GRM5, GRIA1, GRIA2, GRIN1*, and *GRIN2B*) and glutamate synapse scaffolding genes SH3 And Multiple Ankyrin Repeat Domains 2 (*SHANK2*) and Homer Scaffolding Protein 2 (*HOMER2*). Of these newly identified transcripts, GRIN1 and *SHANK2* expression had a positive significant correlation with total song. Overall, two different *in silico* analyses revealed that the glutamate system is heavily involved in the production of gregarious song in starlings.

## Conclusions

Brandon Polzin and colleagues [13] have now established that conserved neurotransmitter systems, such as glutamate and dopamine pathways, are fundamental to producing gregarious song behavior in birds (Fig. 1). As singing is a rewarding behavior, the observation that dopamine receptor transcripts are highly expressed in the preoptic area provides a clear substrate for the motivation to engage in song. The findings reveal a dual pathway exists in the mPOA for the motivation to produce song. Testosterone signalling in the mPOA drives song production associated with increased animal fitness involved in breeding (i.e., mate attraction, territorial defense) and neurotransmitter systems including glutamate and dopamine (via *DRD2*) facilitate social cohesion via gregarious song. Overall, the paper provides a comprehensive dataset that can be mined to uncover common, evolutionary ancient molecular pathways that contribute to the neural control of highly social behavior in vertebrates.

## Data Availability

N/A.

## Abbreviations

Alpha: amino-3-hydroxy-5-methyl-4-isoxazole propionate (AMPA)
CHAT: Choline O-acetyltransferase
cDNA: Complementary DNA
DRD: Dopamine receptor
GABA: Gamma-aminobutyric acid
GRIN1: Glutamate ionotropic receptor N-methyl-D-aspartate type subunit 1
GRM5: Glutamate metabotropic receptor 5
GnRH: Gonadotropin-releasing hormone
HOMER2: Homer Scaffolding Protein 2
mPOA: Medial preoptic area
MEGENA: Multiscale embedded gene co-expression network analysis
CHRM4: Muscarinic acetylcholine receptor 4
SHANK2: SH3 And Multiple Ankyrin Repeat Domains 2
